# Preferred techniques of hair harvest for medical testing among adult pulmonary TB patients

**DOI:** 10.4314/ahs.v23i4.6

**Published:** 2023-12

**Authors:** Grace Muzanyi, David K Mafigiri, Robert Salata, Moses Joloba, Jackson Mukonzo, Mohammad Ntale, Paul Mubiri, Godfrey Bbosa

**Affiliations:** 1 Department of Pharmacology and Therapeutics, Makerere College of Health Sciences; 2 Uganda-Case Western Reserve University Research Collaboration; 3 Makerere University School of Public Health; 4 Case Western Reserve University, Cleveland Ohio; 5 Makerere University School of Social Sciences; 6 Makerere University School of Biomedical Sciences; 7 Department of Chemistry, College of Natural Sciences, Makerere University

**Keywords:** Tuberculosis, hair harvest technique, hair medical testing

## Abstract

**Background:**

Antiretroviral hair drug levels are currently being used to monitor adherence to HIV treatment. There is currently a dearth of literature on the preferred technique(s) of hair harvest for medical testing in the context of African multicultural settings.

**Objective:**

To explore the preferred techniques(s) of hair harvest for medical testing among TB patients.

**Methods:**

We used a descriptive phenomenological approach to conduct interviews for 15 TB patients from the 06^th^ through the 24^th^ of June 2022. Data was organized by N-VIVO version 10 and analysed step by step using a thematic analytical approach.

**Results:**

Participants aged <30 years were more knowledgeable, positively perceived, and experienced about the salon-based hair cutting technique compared to those aged≥30 years old. Participants aged ≥30 had experience, flexibility to use, and were knowledgeable in all three techniques, Overall, for all age categories (<30,30-40 and >40 years), majority of the respondents were knowledgeable, flexible and experienced in using all the three techniques.

**Conclusion:**

The majority of TB patients were knowledgeable, experienced and flexible about the hair cutting techniques however, efforts are needed to educate the youth that hair for medical testing can be cut by any of the three techniques without changing their cosmetic look.

## Introduction

Tuberculosis (*TB*) has remained a global public health problem albeit the interruption in the diagnostics by the Covid pandemic^[i]^ . According to WHO, Worldwide, TB is one of the top 10 causes of death, and the leading cause of death from a single infectious pathogen, mycobacterium tuberculosis complex above human immune virus (HIV)/acquired immune deficiency (AIDS). Millions of people continue to develop the disease worldwide annually 1

The current first-line treatment for drug-sensitive tuberculosis is a combination of 4 drugs (E, R, H, and Z) in the intensive phase (2 months) and R+H in the continuation phase (4 months) 2 but the duration of treatment can be prolonged to beyond 6 months for patients who don't culture convert by the end of the intensive phase and have cavities on a pre-treatment chest radiograph. Drug-resistant cases are managed with a second-line regimen and individualized regimens based on drug susceptibility testing and treatment lasts longer (ranging from 9 months to 2 years) 3 than drug-susceptible TB. This long duration of treatment exacerbated by the high pill burden is very inconvenient to patients and is plagued by high rates of default.

Adherence questionnaires and pill counts that are frequently used to quantify TB treatment 4 adherence is limited by parental/guardian recollection, the frequent provision of answers deemed socially acceptable, and the low accuracy and reliability of pill counts. However, these shortcomings can be overcome by a directly observed therapy strategy, but this strategy is insufficient to assess adequate drug exposure. Single or multiple plasma drug levels 5 are generally used for assessing drug exposure, but these measures only represent a small window of exposure and has had inconsistent success in predicting treatment outcomes. Furthermore, plasma drug concentrations can show high rates of intra-individual variability. Plasma trough levels of short half-life drugs are likely to be undetectable and cannot give information in PK-PD analyses about drug exposure over time. Furthermore, some TB drugs such as isoniazid are unstable in stored plasma samples 6. An alternate, preferably non-invasive, method of assessing drug levels would provide an important tool for the evaluation of drug exposure and adherence.

Monitoring drug levels in hair samples have been applied in a myriad of diseases such as HIV 7 and epilepsy 8 to assess both adherence and exposure since drugs incorporate into hair from the plasma over weeks to months. Harvest of human hair from the body of patients is a very sensitive issue in Africa given the cultural and faith complexity of the African society 9, and the witchcraft beliefs attached to certain body parts 10. Hair can be harvested from the scalp of patients using different techniques including razor blades, scissors, or hair-cutting electrical machines. In the far past, hair cutting from the body was done by razorblades and in the intermediate past, scissors were introduced. In recent times, the introduction of modern electronic hair-cutting machines has revolutionized hair care and it's the method mostly used in today's salons especially when it comes to bringing out the desired cosmetic look 11. Hospitals' starting to use hair for medical testing is a novel approach, however, the preferred method of hair harvest might differ depending on one's age, religious or cultural background 12. Studies are ongoing to provide data and formulate tools that will start using hair for medical testing especially monitoring adherence to treatment. When such new policies are effected in the medical world, we need to be sure they are acceptable to the patient Communities. To ensure the acceptability of medical hair testing, one needs to explore the patient's preferred techniques of hair harvest. We, therefore, conducted a qualitative study to determine the preferred methods of hair harvest for medical testing among pulmonary TB patients.

## Methods

### Study design

We used a descriptive phenomenological approach 13 to explore the preferred method of hair harvest for medical testing from the body of TB patients with a focus on three methods: the salon-based modern hair-cutting machine, scissors, and razor blade. Participants expressed their views through in-depth interviews.

### Study setting

This study was carried out at the Uganda Case Western Reserve University Research Collaboration TB project clinic within Mulago Hospital, Kampala, Uganda's largest national referral hospital. The TB project clinic is a specialized TB, and HIV research clinic where patients residing within a radius of 30km are screened and enrolled. The catchment area includes places like Wakiso, Mukono, Mpigi, and Kampala.

### Participants and recruitment

We included participants who were confirmed TB patients (Genexpert+/smear+), who had already started TB treatment, and with experience in cutting hair from their bodies for general cosmetic and other purposes. Participants were recruited by the Principal Investigator (PI) through screening at the local TB clinic at the Uganda-Case Western Reserve University Research Collaboration. We excluded TB patients who mentally incapacitated to go through an interview.

A purposive sampling strategy was used to recruit 15 TB patients who were able to provide in-depth information regarding methods of hair cutting from the human body. This sampling strategy was used in this phenomenological approach as it allows the selection of TB patients with a rich source of knowledge on hair-cutting techniques.

### Data collection

All interviews were conducted in person and audio recorded. The interviews were conducted in Luganda, the local language most widely spoken in the catchment area of the Uganda Case Western Reserve University TB project clinic. The Luganda language interviews were then transcribed by the interviewer into Microsoft word followed by a translation into English by an independent experienced qualitative research assistant. The English transcript was then back-translated into Luganda by the interviewer who had conducted the original interview. Any differences in opinion or sections that needed consensus about meaning and representation were discussed and reviewed together with the PI. Data collection continued until saturation was achieved to ensure reliability, conformability, and mitigation of bias in the delineation of the participant's preferred technique of hair harvest for medical testing. The study was conducted in line with the consolidated criteria for reporting qualitative studies (COREQ). The areas covered in the interviews were :(i) participants' experience in cutting hair from their bodies. (ii)Perceptions about hair-cutting techniques. (iii) Preferred method of hair cutting and why.

### Data analysis

N-Vivo version10 software was used to organize the data. A thematic analytical approach 14 was adopted was used to analyse data step by step. We initially did data familiarization and writing of familiarization notes. This was then followed by systematic coding and generating initial themes from collated and coded data. Coding was carried out by two independent experienced research nurses with the third independent nurse acting as the tiebreaker in case of code differences between the two main coders to ensure consensus. The independent nurses were not involved in conducting the interview. About 80% of the emerging codes were similar. The principal investigator and the three independent nurses continued developing and reviewing themes, refining, defining, and naming themes until central themes emerged. This step was repeated at least four times until a theme was eventually settled on. This was then followed by writing a report.

### Ethical considerations

This study was approved by the ethics committee of the Makerere University School of Biomedical Sciences (SBS-2021-18) and the Uganda National Council for Science and Technology (HS2231ES). The researchers ensured that all participants were taken through the informed consent, given ample time to think through, consulted were needed, and allowed to make an informed decision whether to participate or not. All consenting participants signed the informed consent form. The researchers ensured participant confidentiality and assured the participants that the findings will be published anonymously.

## Results

Fifteen TB patients participated in the in-depth interviews. The median age was 30.0(IQR=14.0) and 53% of the participants were male (Table 1).

**Table 1:** Characteristics of study participants(N=15)

Variable	Frequency	Percentage
**Gender**		
Male	8	53.33
Females	7	46.67
Transgender	0	0.00

**Age**		
18-30years	8	53.33
30-40years	4	26.67
>40years	3	20.00

**Education level**		
< =Primary 7	6	40.00
S.1-S.4	5	33.33
S.5-S.6	1	6.67
>S.6	2	13.33
No education	1	6.67

**Duration of TB** **Symptoms**		
0-3months	11	33.33
4-6months	2	13.33
>6months	1	6.67
Missing	1	6.67

**How do you usually cut** **your hair on the head**		
Machine	5	33.33
Scissor	2	13.33
Razorblade	2	13.33
Any of the three	5	33.33
I don't know	1	6.67

The results delineate a phenomenon of outpatient TB patients who are already on TB treatment with a life experience of haircuts from their bodies. The thematic analysis yielded 3 main themes with the first one being participants' perceptions about cutting hair using any of the three techniques (the modern hair-cutting machine, scissors, and razor blades), theme 2 was perceptions on the preferred method of cutting hair given the options of the modern machine, scissors, and razor blade and finally, theme 3 was descriptions of prior experience in cutting hair for medical testing.

### Theme 1: Perceptions about using the modern hair-cutting machine, scissors, and razor blades

Participants described their perceptions on the methods of hair cutting with the young age group (18-30yrs) expressing more views on the modern salon-based hair-cutting machines but seemed to have fewer views on the scissor and razorblade methods.

One participant described

*“I have always used the machine in saloons to cut my hair. I feel it is odd to use a scissor or razor blades. I wonder how one's hair will look if you don't use a machine”*

The older age groups (age 30-40 and >40yrs) had views on all the methods compared to the young age group (age 18-30yrs) who mainly expressed views on the modern hair-cutting machine.

One participant described

*“I have no problem with any of the three. I have used a razor blade and scissors before to cut my hair. I currently use the machine. I didn't get any problem with any of the three”*, Said a 46-year-old female

### Theme 2: Perceptions of the preferred technique of hair cutting in the context of the modern machine, scissors, and razor blades

The young age group (<30 years) expressed a preference for using the modern salon-based hair-cutting machine but overall, with all age groups combined majority patients expressed the fact that they were free to use any method. A few of the participants, however, expressed a dislike for sharing machines as they stand the risk of infection.

One participant described:

*“If the hair is harvested the way it is cut in salons where they use the same machine to cut different people, one can easily get infected with diseases”*. Said a 24-year-old male

**Figure 1:**
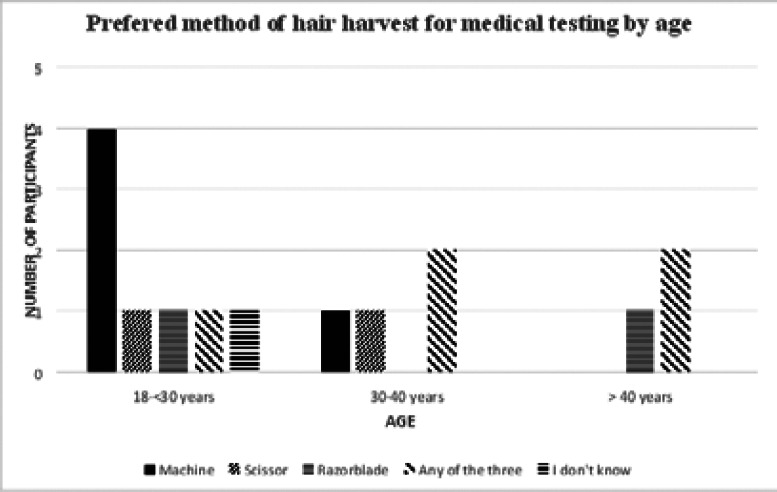


Older age groups (figure 1) preferred any of the three methods.

### Theme 3: prior experience in cutting hair for medical testing

Majority of the patients interviewed expressed having experience in cutting their hair for cosmetic purposes but not for medical testing. They were hearing about using hair for medical testing for the first time and a few were surprised to hear this approach. They felt it was going overboard to start using hair for medical testing. Participant described

*“Using hair in hospitals for medical tests is something too much. I have never even heard about it. It is going overboard. Someone taking your hair can have the intention to harm you in witchcraft?”* said a 38-year-old male

## Discussion

Qualitative research on the use of hair for medical testing is a new approach in the Ugandan setting but important for improving future treatments in certain disease conditions including but not limited to HIV, Tuberculosis, epilepsy, hypertension, etc. The main themes in our study; were the participant's perception of each of the methods for cutting hair, their perception of the preferred method of hair harvest, and prior experiences of hair cutting for general cosmetic purposes, etc.

The first theme identified in our study was the participant's perception of each of the three methods; modern salons like hair cutting machines, scissors, and razor blades. Young participants delineated and had much more information on the salon-based hair-cutting machines as compared to the scissor and razor blades. This could be attributed to the fact that youth love taking care of their hair more than their adult counterparts and this preference compels them to visit the salon more for different hairstyles. The older group (>30 years) expressed knowledge of all three methods of the machine, scissors, and razor blades. This could be attributed to the fact the older age group could have used any of the scissor or razorblade methods which were common in the past compared to the youth who are only exposed mainly to the salon-based hair cutting machine Myoung-Hee et al 15 did a Study on Image Perception according to Perceiver's Social Value and Hair Style Variation. He found that social value, hairstyle, and hair colour are significant characteristics when perceiving women's image however; this study did not address perceptions of the different hair-cutting techniques as our study did.

Nayak BS et al 16 did a Study on scalp hair health and hair care practices among Malaysian medical students. She found that since most students colour their hair and employ various hairstyling methods, they should be educated regarding the best hair care practices to improve their scalp hair condition and health. This study was close to our research question but still did not address the perceptions of the students on the different hair-cutting methods.

In the currently published literature, no study has directly addressed this research question on the perceptions of people about the different methods of hair harvest. Our study is the first one to explore this area.

The second theme identified in our study was the perception of the actual preferred method of haircutting. Still, the young age group expressed a preference for the modern salon-based hair-cutting machine over a scissor or razor blade. Many delineated the salon-like machine as something they have been seeing since childhood and it is therefore deemed safe and effective as compared to the other methods they have never seen before. The older age group was more flexible and the majority expressed ease of using any of the three methods of the machine, scissors, or razor blade. Park Jang Soon etal 17 did a study on the correlation of demographic characteristics of Korean mean and the preference of women's grading such as age, education, marital status, occupation etc. They investigated the preference of men for women's graduation haircuts. It was found that majority of the men preferred the longer graduation hair cut for women. He also found that a statistically significant difference was found in the behaviour of haircuts by age, with natural and easy-care voluminous hairstyles being preferred. Second, according to an analysis of preferences by head point using hairstyle preferences by age as the age of hairstylists became younger, repetitive one-length haircuts were more preferred. As age became older, in contrast, changes in hair length and layered haircuts were more preferred. Third, in an analysis of preference of haircut technique by experience as well, as hairstylists were more experienced, more diverse techniques were preferred. This study is in agreement with our findings where the older age groups were flexible with any technique of haircuts. This study came is close to the finding s in this theme but didn't address the preferred techniques for haircuts directly.

The third theme identified in our research was prior experiences of cutting hair for medical testing. The majority of the participants expressed no prior experience with cutting hair for medical testing. This was consistent across all three age groups. They expressed that this is a new approach that they have never heard of or gone through. They expressed serious reservations and felt it was too much for one to start using hair for medical testing. Several studies have been conducted on HIV 7, MDR TB 18, and epilepsy 8 in the use of hair for medical testing. Our study is the first to explore this theme of prior experience using hair for medical testing in the Uganda setting and this explains the patient community's lack of experience with this type of medical testing.

The strength of our study is that it was carried out in the typical traditional African urban setting where there are lots of different hair care styles and differing methods of haircuts.

The weakness of our study is that we didn't probe for specific economic status affiliation per participant which could confound some of the responses we witnessed in the interviews.

## Conclusions

The main aim of this research was to explore the preferred method of hair harvest for medical testing among pulmonary TB patients in the context of the participant's lived experiences, culture, and perspectives. Young participants interviewed in this study preferred the salon-like hair-cutting machine to be used for hair harvest for medical testing. The older group preferred any of the three methods; machine, scissor, or razor blade but overall, the participants were free with any of the three methods.

## Policy implications

1. There's a need to develop a plan to sensitize the general public, especially the youth that hair can be used to do routine hospital testing and can be harvested using a scissor/razorblade in the hospital without changing their looks.
2. Based on our findings, further qualitative research is needed to examine these themes for generalization to prepare TB patients especially the youth and the general patient community for future tests involving hair across different disease spectrums.
